# A Novel ENU-Mutation in Ankyrin-1 Disrupts Malaria Parasite Maturation in Red Blood Cells of Mice

**DOI:** 10.1371/journal.pone.0038999

**Published:** 2012-06-19

**Authors:** Andreas Greth, Shelley Lampkin, Preethi Mayura-Guru, Fleur Rodda, Karen Drysdale, Meredith Roberts-Thomson, Brendan J. McMorran, Simon J. Foote, Gaétan Burgio

**Affiliations:** 1 The Menzies Research Institute of Tasmania, University of Tasmania, Hobart, Australia; 2 Australian School of Advanced Medicine, Macquarie University, Sydney, Australia; Burnet Institute, Australia

## Abstract

The blood stage of the plasmodium parasite life cycle is responsible for the clinical symptoms of malaria. Epidemiological studies have identified coincidental malarial endemicity and multiple red blood cell (RBC) disorders. Many RBC disorders result from mutations in genes encoding cytoskeletal proteins and these are associated with increased protection against malarial infections. However the mechanisms underpinning these genetic, host responses remain obscure. We have performed an N-ethyl-N-nitrosourea (ENU) mutagenesis screen and have identified a novel dominant (haploinsufficient) mutation in the *Ank-1* gene (*Ank1^MRI23420^*) of mice displaying hereditary spherocytosis (HS). Female mice, heterozygous for the *Ank-1* mutation showed increased survival to infection by *Plasmodium chabaudi adami* DS with a concomitant 30% decrease in parasitemia compared to wild-type, isogenic mice (wt). A comparative *in vivo* red cell invasion and parasite growth assay showed a RBC-autonomous effect characterised by decreased proportion of infected heterozygous RBCs. Within approximately 6–8 hours post-invasion, TUNEL staining of intraerythrocytic parasites, showed a significant increase in dead parasites in heterozygotes. This was especially notable at the ring and trophozoite stages in the blood of infected heterozygous mutant mice compared to wt (p<0.05). We conclude that increased malaria resistance due to ankyrin-1 deficiency is caused by the intraerythrocytic death of *P. chabaudi* parasites.

## Introduction

Malaria is caused by transmission of the protozoan parasite Plasmodium, and kills almost 1 million people annually, and affects a further 300–500 million (WHO 2010). Multiple epidemiological studies in human populations have shown that host genetics is a major determinant in the susceptibility to malaria infection. [1]–[3]. Numerous variants and mutations have been identified that associate with survival and impaired parasite growth, including several polymorphisms in genes encoding erythrocyte-expressed proteins [4]–[8]. For example the erythrocyte Duffy antigen receptor for chemokines (DARC), encoded by the *FY* gene, is necessary for the invasion by *P. vivax* of RBCs [9]–[11]. A mutation in the GATA motif of the *FY* promoter prevents erythrocyte expression of FY. This mutation is common in African populations and confers protection against *P. vivax* infection [12].

The cytoskeleton of erythrocytes has been a major interest in red cell biology for decades as multiple disorders arise from mutations in cytoskeletal components [13]. Several such red cell disorders are also associated with resistance to malarial infection [14]–[18]. For example, individuals with hereditary elliptocytosis have deficiencies in either protein 4.1 or glycophorin C and have been reported to show resistance towards malaria infections [19]. Other RBC disorders such as hereditary spherocytosis (HS) [13], [20] have also shed light into the host-parasite interaction due to their involvement in malaria pathogenesis [21], [22]. However the elucidation of the mechanisms of resistance mediated by changes to these host erythrocytic proteins remains incomplete.

The murine host response to a malarial infection is faithful to the human response to human malarias [23]–[26]. Mouse models of malaria have been used to study genetic factors that determine the host response to infection and to identify novel mechanisms that confer protection against Plasmodium [27], [28]. Similar to humans, there is considerable variability in either the rate of development of blood parasitemia or outcome to infection in different inbred mouse strains [29]–[31]. Beside the identification of several quantitative trait loci that determine susceptibility towards rodent malarial infection, investigation of various mouse mutants have revealed novel genes associated with malaria pathogenesis [24], [30]–[33]. Given the difficulty identifying genes underpinning quantitative trait loci, we have employed a complementary approach using N-ethyl-N-nitrosourea (ENU) to generate random point mutations in mice to identify novel genes and mechanisms that underlie susceptibility to malaria infection. This strategy has been previously employed to decipher the genetic architecture determining several phenotypes [34], [35] including other infectious diseases [36]-[38]. In our ENU mutagenesis screen, we aimed to identify novel mutations that increase the resistance of an otherwise susceptible strain of mouse to a malarial infection. We describe here an ENU-mutant line of mouse with increased survival to *P. chabaudi adami* DS infection, carrying a novel hypomorphic mutation in the *Ank-1* gene (*Ank1^MRI23420^*).

Ankyrin-1 is a large (210 kDa) cytoskeletal protein encoded by the *Ank-1* gene and is found predominantly in the erythrocyte membrane. Mutations in human *Ank-1* have been studied for their role in the inherited hemolytic anaemia disorder, HS [20]. Over 50% of HS cases are caused by mutations in this gene [39]. Recently, *Ank-1* has been investigated for its role in malaria infections using a naturally occurring mutant (*Ank-1* ^nb^) [17] and an ENU generated mutant (*Ank-1* ^1674^) mouse line [14]. Both of these mutations result in an abnormal ankyrin-1 protein in the erythrocyte. Mutant mice carrying these mutations are more likely to survive infections from *P. chabaudi* and *P. berghei* [14], [17]. However the role of mutant ankyrin-1 in conferring increased resistance remains a point of conjecture. Studies have variously suggested that merozoite release, [40]–[42] parasite invasion [17] and intraerythrocytic development [17] may be affected, although the evidence for these is not conclusive.

In this study we aimed to further characterise the mechanistic basis of malarial resistance imparted by ankyrin-1 deficiency through the analysis of the Ank1^MRI23420^ mouse line. We present novel evidence that maturation of intracellular *P. chabaudi* parasites is impaired in ankyrin-1 deficient RBCs. Although we cannot exclude other contributions such as splenic clearance we conclude that parasite growth retardation is the major mechanism contributing to the resistant phenotype observed in *Ank-1^MRI23420/+^* mice.

## Results

### Identification of a Novel ENU *Ank-1^MRI23420^* Mutation

The ENU mutant line MRI23420 was generated during a screen for dominant ENU-induced mutations that affect RBCs in an inbred line of mice, SJL/J. Full automated blood analysis was conducted on samples collected from G1 animals at 7–8 weeks of age. The line MRI23420 was identified based on a mean corpuscular volume (MCV) that was 3 standard deviations less than the normal population (MCV = 45.2±0.2 fl in mutant versus MCV = 52.0±0.4 fl in wild type mice; p-value <0.001) (Table 1).

**Table 1:** Haematological parameters on wild-type and Ank-1^MRI23420/+^ mice.

	*WBC*	*RBC*	*HGB*	*HCT*	*MCV*	*MCH*	*MCHC*	*RDW*	*PLT*	*%Retic*
Wild-type	6.1±0.1	5.1±0.1	70.4±1.7	0.25±0.01	53.4±0.2	15.7±0.5	293.9±8.3	15.4±0.1	498.3±7.8	2.5±0.2
Ank-1 ^MRI23420/+^	6.1±0.2	5.5±0.1**	72.0±1.4	0.26±0.02	45.3±0.1**	13.6±0.3**	285.7±5.9	15.5±0.1	540.6±13.4*	3.0±0.2

Automated full blood analyses were obtained on 69 wt and 72 *Ank-1^MRI23420^* mice at 7 weeks of age. Values are represented as mean value ±SEM. Statistical differences are indicated as (*) p-value <0.05 and (**) p-value<0.001. WBC indicates white blood cell count; RBC, red blood cell count; HBG, hemoglobin; HCT, hematocrit; MCV, mean corpuscular volume; MCH, mean corpuscular hemoglobin; MCHC, mean corpuscular hemoglobin concentration; RDW, red cell distribution width; PLT, platelet count; and %Retic, %Reticulocytosis.

**Figure 1:**
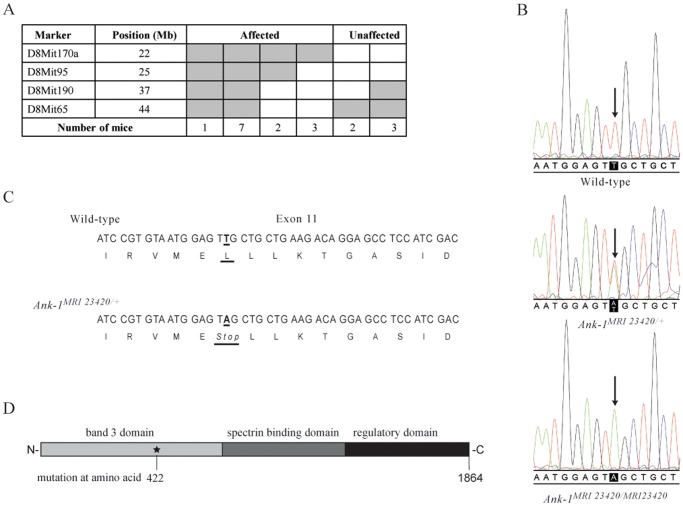
Identification of the Ank-1MRI23420 allele. (A) Haplotypes of 2nd generation offspring. Narrowing down critical interval using microsatellites on chromosome 8. Grey boxes represent heterozygous and white boxes homozygous mice. The candidate interval for Ank-1MRI23420 was refined to 22–25 Mb on Chr. 8. (B) DNA sequence electropherograms showing the T to A transversion in exon 11 for wt, Ank-1MRI23420/+ and Ank-1MRI23420/MRI23420 mice. (C) Schematic view of the amino acid change. (D) Representation of the position of the mutation within the protein.

The mutation was mapped by backcrossing SJL/J mice exhibiting the mutant phenotype to C57BL/6 animals. Using an affected-only mapping strategy, progeny (F1s & N2s) were selected on the basis of an MCV <50.0 fl and DNA analysed using genome-wide SNP genotyping (20 affected N2 mice using 300 polymorphic SNPs). Linkage analysis identified a region on chromosome 8 shared by all affected N2 mice (LOD  = 5.11), and the critical interval was further defined using microsatellite mapping to a region 22 - 25 Mb from the centromere (Fig. 1A). Within this interval, the *Ank-1* gene was selected as a candidate and sequencing the affected G1 founder mouse revealed a single heterozygous mutation, which was also present in the affected N2 animals. The mutation, a single point transversion (T A) in exon 11 at nucleotide position 1265 of *Ank-1* (Fig. 1B), creates a premature termination codon at position 422 predicted to produce an ANK-1 protein truncated within the band 3 binding domain (Fig. 1C &1D).

**Figure 2:**
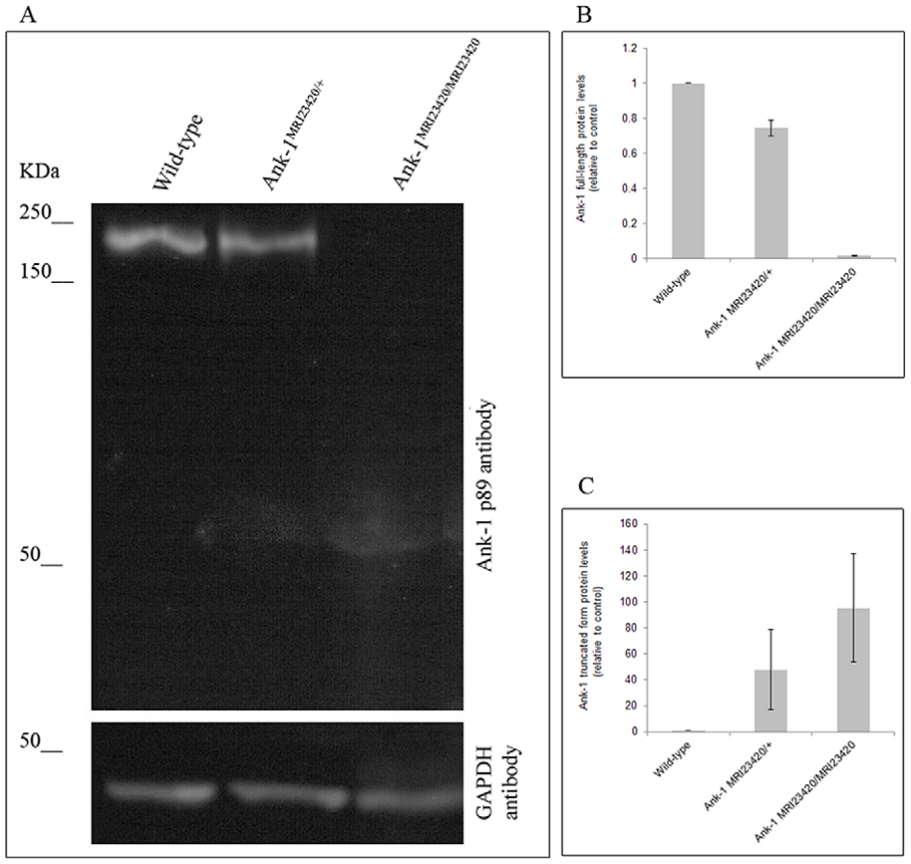
Protein levels of ankyrin-1 analysis. (A) Immunoblot of erythrocytic ankyrin-1 in wt, Ank-1MRI23420/+, and Ank-1MRI23420/2340 mice with the N-terminal antibody ANK-1 p89. Quantification of the intensity of bands by densitometry for full-size ankyrin-1 (210 kDa) (B) and the truncated species (C) (calculated size 49 kDa). Error bars indicate SEM.

To investigate the effect of the mutation on *Ank-1* expression, we conducted immuno-blotting on whole blood lysates from wt, *Ank-1^MRI23420/+^* and *Ank-1^MRI23420/MRI23420^* mice. A 210 kDa anti-mouse ANK-1 immunoreactive band, corresponding to the full-length protein, was observed in samples from the wt and heterozygous mice, while this band was not detected in the homozygote mice (Fig. 2A and 2B). A smaller band (∼50 kDa) was also present in the heterozygous and homozygous mutant samples, closely matching the theoretical size of the truncated form of ankyrin-1 (49 kDa; Fig. 2A). Quantification of these immunoreactive bands revealed reduced levels of the full-length protein in *Ank-1^MRI23420/+^* blood cells compared to wt cells, while *Ank-1^MRI23420/MRI23420^* cells displayed a greater amount of the truncated form compared to their heterozygous relatives. To assess if a compensatory mechanism substitutes for the lack of functional protein, a quantitative PCR was conducted using two *ANK-1* primers spanning before and closely after the ENU mutation (Fig. S1A and S1B). Relative quantification of *Ank-1* mRNA expression in different tissues found significantly elevated levels of transcript in some *Ank-1^MRI23420/+^* tissues. In the spleen and in the brain, the transcript level of *Ank-1^MRI23420/+^* is respectively 4 and 2.5 fold over expressed and 0.7 fold under expressed in the kidney. No differences were found on transcripts level in the liver.

### The ENU Blood Mutant MRI23420 Displays an Hereditary Spherocytosis Phenotype

**Figure 3:**
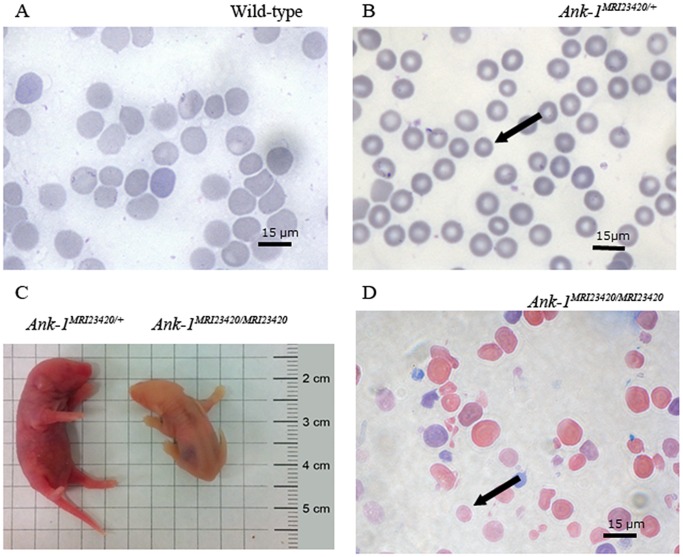
Identification of *Ank-1^MRI23420/+^* and *Ank-1^MRI23420/23420^* mice. Giemsa stained peripheral blood smears from (A) wt, (B) *Ank-1^MRI23420/+^,* and (D) *Ank-1^MRI23420/MRI23420^* mice. Black arrows indicate microcytic RBC in *Ank-1^MRI23420/+^* and spherocytes in *Ank-1^MRI23420/23420^*. (C) Jaundiced postnatal day 1 of *Ank-1^MRI23420/MRI23420^* pup and an *Ank-1^MRI23420/+^* control littermate.

The observed frequency of 10% live births of homozygous littermates compared to an expected 25% suggesting that mice homozygous for the mutation are embryonic or neonatal lethal. *Ank-1^MRI23420/MRI23420^* mice displayed severe jaundice and had a life expectancy of only 12–48 h (Fig. 3C). Autopsy revealed distinct splenomegaly (spleen weight divided by body weight in wt mice 5.5±0.4 g versus 7.1±0.6 g in *Ank-1^MRI23420/MRI23420^*, p-value <0.05) and Giemsa stained blood smears showed distinct amorphous poikilocytosis with marked spherocytes, severe RBC fragmentation, and increased immature RBCs (Fig. 3D). Due to the severity of their phenotype, homozygote mice were not subject to malarial studies.

**Figure 4:**
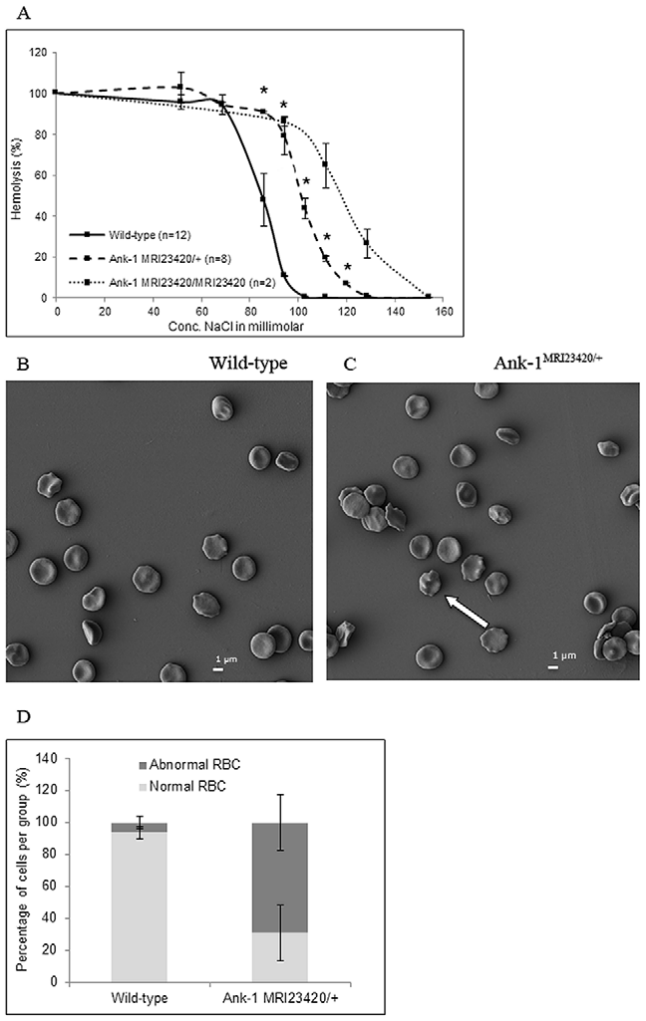
Hematological phenotype of *Ank-1^MRI23420/+^* RBCs. (D) Osmotic fragility plot of wt, heterozygous and homozygous mice. Scanning electron microscope imaging of peripheral blood from (B) *Ank-1^MRI23420/+^* and (C) wt uninfected mice. The white arrow indicates erythrocyte membrane blebbing and deformity. (D) Enumeration of the proportion of normal and abnormal RBCs observed with error bars indicating SEM.

Heterozygous *Ank-1^MRI23420/+^* mice displayed a slight splenomegaly (0.11±0.03 g for wt mice versus 0.16±0.01 g for heterozygous mice; p-value <0.05). Histological examination revealed changes in the splenic architecture with a reduced medulla and a proliferation of the extramedullary compartment (Fig. S2A and S2B). In addition, Perl’s blue Prussian staining revealed extramedullary iron deposition (Fig. S3A and S3B). Quantification of non-heme iron (Fig. S3C) demonstrated an almost 2 fold increase in iron deposition in the spleen of heterozygous mice consistent with an iron overload phenotype. Hematological analysis of the heterozygous mutant mice revealed a number of red cell abnormalities (Table 1) including a marked microcytosis and mild polycythemia. This was confirmed in peripheral blood smears, where small, hyperchromic red blood cells were observed (Fig. 3A,B). Scanning electron microscopy imaging revealed that over half (68.9±17.4%) of the red cells in *Ank-1^MRI23420/+^* mice were severely deformed with obvious membrane blebbing, although no fragmentation was seen. By comparison wt controls exhibited 6.25±3.95% of abnormal RBCs (Figure 4B-D). To investigate this further, we conducted osmotic fragility assays and found that cells from heterozygous mice lysed at significantly higher salt concentrations than wt (Fig. 4A). In concert with the observed anaemic phenotype we observed an almost two-fold increase in serum levels of the erythropoietin, the hormone controlling erythropoiesis in *Ank-1^MRI23420/+^* mice (530.7±47.7 pg/ml; p-value <0.001) compared to wt (278.3±24.9 pg/ml). Several cases of *Ank-1* mutations in both human and mice have been reported in the literature and all of them have been associated with a HS phenotype exhibiting anaemia, splenomegaly, and a higher osmotic fragility in RBCs [14], [39], [43], [44].

### *Ank-1^MRI23420/+^* Mice are Resistant to Malaria Infection

**Figure 5:**
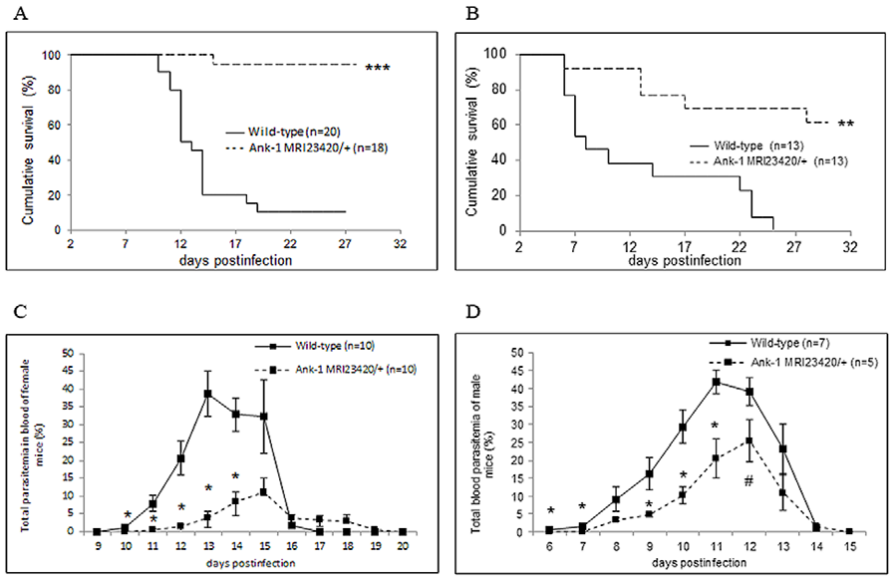
*Ank-1^MRI23420/+^* mice display resistance towards malarial infections. Kaplan Meier survival curve for (A) female and (B) male animals. Number of infected RBCs in (C) female and (D) male mice. Infection dose was 4×10^4^ and 2×10^4^ for female and males respectively. Error bars indicate SEM and statistical differences are marked with a students t-test p-value <0.05 (#), p-value <0.01 (*), and Mantel Cox Log rank with p-value <0.001 (**) and p-value <0.0001 (***).

We sought to characterise the effect of the *Ank-1^MRI23420^* mutation in a malarial infection. Heterozygous and wt mice of both gender were challenged with a normally lethal dose of *P. chabaudi adami* DS. A dramatic increase in survival of the *Ank-1^MRI23420/+^* mice compared to wt SJL was observed in both males and females (p<0.001 and p<0.0001, respectively, using a Mantel-Cox test); the respective mortality rates for each sex were 6% (females) and 38% (males) for *Ank-1^MRI23420/+^* and 90% (females) and 100% (males) for SJL mice (Fig. 5A-B). Further we monitored the development of blood parasitemia. The proportions of infected RBCs in mutant animals of both sexes peaked at a significantly lower level and later time (one or two days) compared to wt (Fig 5C-D). Therefore as well as acting dominantly to cause an HS-like phenotype, a single copy of the *Ank-1^MRI23420^* mutation also results in a dramatically enhanced resistance to malaria infection.

### The *Ank-1^MRI23420^* Malaria Resistant Phenotype is due to a Red Cell Autonomous Effect

**Figure 6:**
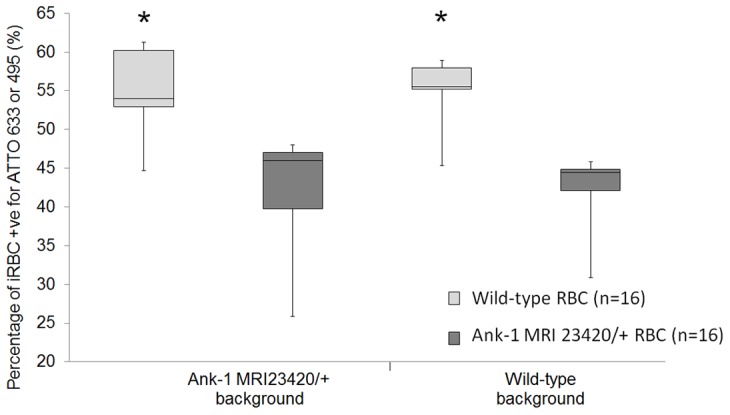
Infection rate of *P. chabaudi* in *Ank-1^MRI23420/+^* compared to wt RBCs is impaired. Uninfected RBCs from wt and *Ank-1^MRI23420/+^* mice were labelled individually with different fluorescent dyes mixed at a 1∶1 ratio and injected into infected animals of both groups. 10 hours later tail blood was taken and analysed on flow cytometry gating for infected RBCs +ve for either dye (ATTO 633 & 495). Results were calculated as the mean of normal and reversible labelled RBCs from each group for each background separately. Error bars are presented as SEM and (*) indicate statistical differences with a p-value <0.0001.

To assess the contribution of abnormal red cells and possible secondary effects of the mutation (eg. altered splenic function) in the malaria resistant phenotype, we developed an *in vivo* parasite invasion and growth assay. RBCs isolated from uninfected wt and *Ank-1^MRI23420/+^* mice were labelled with individual fluorescent markers (ATTO-495 and -633), mixed in equal proportions and injected back into the bloodstream of infected recipient mice of either genotype (wt and *Ank-1^MRI23420/+^*). Following a period to allow for parasite reinvasion, samples were collected and examined for the proportion of infected cells of each labelled population growing in animals of each genotype (Fig. S4A-B). The results revealed a significant decrease in the proportion of infected cells from the *Ank-1^MRI23420/+^* line compared to wt (p<0.0001; Fig. 6). This difference was observed irrespective of dye used to label each type of cell, and occurred independently of the genotype of the recipient animal. Therefore, the *Ank-1^MRI23420^* mutation appears to confer the resistant phenotype by directly affecting the ability of the host red to support either invasion, egress and/or growth of the parasite. However, we also observed a modest but significant increase in spleen weight in infected mutant mice around the time of peak parasitaemia (0.30±0.03 g for wt versus 0.82±0.10 g for *Ank-1^MRI23420/+^,* p-value <0.05), consistent with increased splenic clearance of RBCs. We believe that this does not significantly affect the clearance of abnormal cells from the circulation as the proportion of infected cells of either genotype was not dependent on the background. However it is possible that infected cells are cleared more in mutant mice, but this would be similar for both mutant and wt cells. This observation may also be related to the underlying different baseline splenomegaly present in these mice (see above).

### Intraerythrocytic Parasites Die during Infection in *Ank-1^MRI23420/+^* Mice

After merozoites invade RBCs malaria parasites transform into ring forms and subsequently develop into trophozoites that undergo nuclear divisions becoming mature schizonts. In the case of *P. chabaudi* the parasites intraerythrocytic life cycle lasts 24 hours [45] and the parasites show a synchronous growth cycle at low parasitemia.

**Figure 7:**
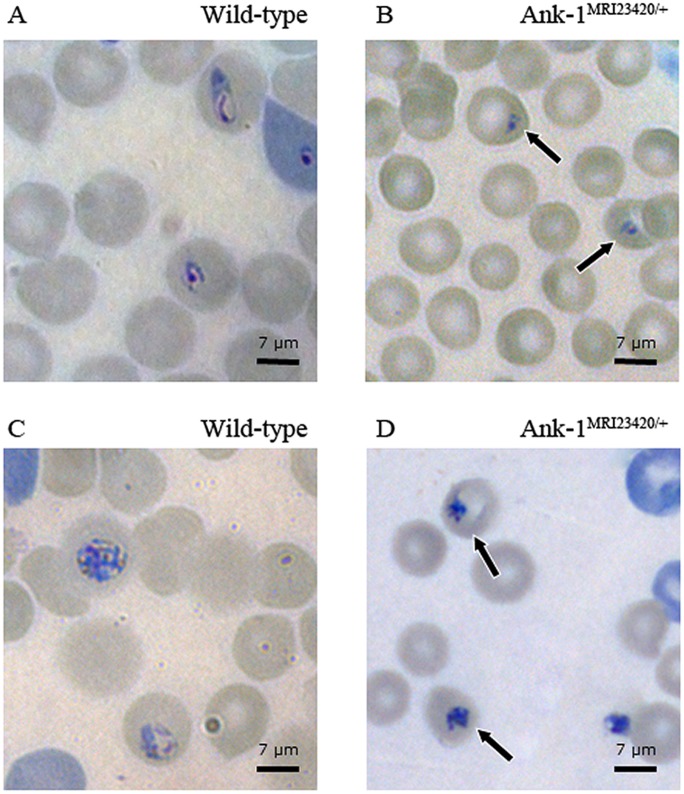
Impaired phenotype of *P. chabaudi* parasites in *Ank-1^MRI23420/+^* RBCs. Giemsa stained blood smears showing the morphology of parasites in wt and *Ank-1^MRI23420/+^* mice 6–8 hours (A-B) and 18–20 hours (C-D) post-invasion. Black arrows indicate condensed phenotype of intraerythrocytic parasites observed in heterozygous mutant mice.

During our observation of the parasites’ asexual life cycle under a light microscope it became noticeable that when compared to wt RBCs, a significant larger proportion of parasites in mutant RBCs were smaller and pyknotic. This phenomenon was visible 6–8 hours (37.4% ±6.3 in *Ank-1^MRI23420/+^* vs. 17.6%±1.1 in wt RBC `s; p-value <0.05) (Fig. 7A and 7B) and 18–20 hours (40.7% 8.0 in *Ank-1^MRI23420/+^* vs. 16.3%±2.2 in wt RBC `s; p-value <0.05 (Fig. 7C and 7D) post-invasion. From this observation we speculated that parasites are less healthy while growing in mutant RBCs and hypothesised that *P. chabaudi* may suffer from retarded development during the erythrocytic life cycle in *Ank-1^MRI23420/+^* mice. Further we observed that in *Ank-1^MRI23420/+^* compared to wt RBCs parasite replication was noticeably lower after 18–20 hours post-invasion (Fig. S5).

**Figure 8:**
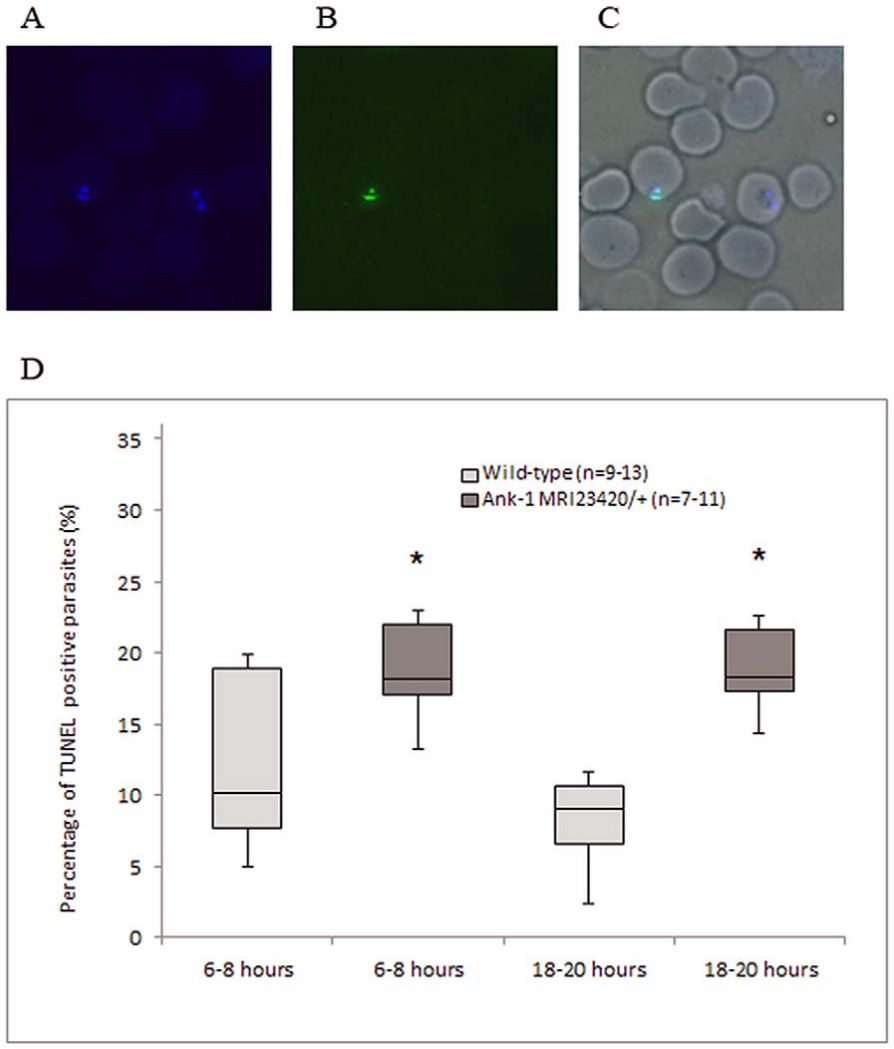
Evidence that maturation of *P. chabaudi* in *Ank-1^MRI23420/+^* RBCs is impaired. (A) Nuclear yellow staining of parasite DNA. (B) TUNEL staining of fragmented parasite DNA, and (C) merged TUNEL and nuclear yellow staining superimposed on brightfield showing infected RBC. (D) Percentage of double positive for nuclear yellow and TUNEL stained parasites in regards to overall parasitemia in RBCs of both cohorts. Blood was collected 6–8 hours (ring stage) and 18–20 hours after invasion (trophozoite stage). Error bars indicate SEM and statistical differences with a p-value <0.05 were indicated by (*).

To objectively quantify these observations we used the TUNEL stain procedure adapted to detect sheared or fragmented DNA, indicative of dying or dead intraerythrocytic parasites (Fig. 8A-C) [46]. Compared to wt, a significant increase of almost two-fold (<0.05 p-value) in dead parasites was apparent in blood taken from mutant mice approximately 6–8 hours after parasite invasion. The proportion of dead parasites in mutant compared to wt mice remained significantly higher 12 hours later (18–20 hours post-invasion) wt (p-value <0.05) (Fig. 8D). This is consistent with our observation of morphologically compromised parasites in mutant cells at both time points and the lower parasite replication rate noticed after 18–20 hours post-invasion. Together this suggests an increased death rate of parasites during their development within *Ank-1^MRI23420/+^* RBCs.

## Discussion

In this study, we identified a novel ankyrin-1 mutation and characterized several associated hematological phenotypes. Further we investigated the mechanism of malaria resistance seen in these ankyrin-1 deficient mice. For the first time, we demonstrate that a mutation in the *Ank-1* gene decreases susceptibility to malaria by reducing survival of *P. chabaudi* in affected RBCs.

Ankyrin-1 protein is a crucial component of the RBC cytoskeletal complex and its deficiency is responsible for the most common type of human hereditary spherocytosis (HS) [20], [39], [47]. Likewise, mice lacking a functional form of the ankyrin-1 protein also share most of the signs and symptoms of human HS [14], [43], [48]. In this report, we demonstrate that our novel ENU-induced mutation in *Ank-1^MRI23420^* mice displays a clinical phenotype mostly consistent with the HS *Ank1^E924X^* mouse model [43]. It has more pronounced symptoms than either the recessively inherited *Ank1^nb^* mutation [48] and the null mutant *Ank1* ^1674^ [14]. Notably *Ank-1^MRI23420^* homozygous mice exhibit a 100% postnatal death rate with severe signs of jaundice due to massive hemolysis. Heterozygous *Ank-1^MRI^* ^23420/+^ mice display a regenerative anaemia and splenomegaly with iron overload. The closest human counterpart to the *Ank-1* ^23420^ murine mutation is the *Ank-1^Bari^* mutation which has a frame-shifting deletion in exon 12 leading to a truncated protein. Interestingly, and similar to the *Ank-1^MRI23420^* phenotype, humans with the *Ank-1^Bari^* mutation have a more severe phenotype than other human ankyrin-1 mutations [44]. Despite the differences in the phenotypic severity between the murine HS models all shared several hematological symptoms. This includes an increased RBC count, increased osmotic fragility and red cell deformity. Therefore, this novel ENU-induced mutation represents another model for the trial of preclinical therapeutic interventions for the treatment of affected individuals with dominant inherited HS.

Importantly, heterozygous *Ank-1^MRI23420^* mice exhibited a dramatic increase in resistance towards malaria. Previous studies focused on two different *Ank-1* mice carrying different mutations in the regulatory domain of the protein; the normoblastosis mutation (*Ank-1^nb^*) [48] and *Ank-1^1674^* (Rank et al. 2009). Both of these strains exhibit HS and also display a survival advantage to *Plasmodium chabaudi* infection [14], [17]. However, the ENU-induced *Ank-1^MRI23420/+^* mice exhibited a delay in the rise of peak parasitemia and a more dramatic reduction in the height of the peak (at least 20 to 40%) compared to heterozygous *Ank-1^nb^* mice (10%). Increased malaria resistance was initially observed 20 years ago in the *Ank-1^nb^* mice; however the mechanism underpinning malaria resistance in mice carrying ankyrin-1 mutations remains elusive. Rank and colleagues [14] examined several potential mechanisms including a hypothesised impaired invasion mechanism of the parasite into RBCs. Despite their efforts no differences in invasion efficiency or altered RBC survival between mutant and wt was found. The only possible functional difference between *Ank-1^1674^* and wt mice was a significant increase in osmotic fragility in affected RBCs. This could possibly result in an increased clearance of infected mutant cells from the circulation by the spleen.

A postulated impairment of parasite invasion into ankyrin-1 deficient RBCs has received considerable attention. This is not surprising considering that the observed increase in osmotic fragility and deformity in ankyrin-1 deficient RBCs is likely to disrupt an association between the cytoskeleton and invading parasite. In support of this hypothesis, elliptocytic RBCs with protein 4.1 and glycophorin deficiencies have been reported to inhibit *P. chabaudi* entry. In contrast is the study by Rank and colleagues [14] which did not find evidence for this mechanism using an *in-vitro* invasion assay system with *P. berghei* as a parasite model. Nevertheless by injecting a 1∶1 mix of both host fluorescently labelled RBCs into wt and *Ank-1^MRI23420/+^* mice in an *in-vivo* assay system we demonstrated that *Ank-1^MRI23420/+^* RBCs were significantly less likely to be infected than wt in a cell-autonomous manner. So far we cannot conclude from our results if the decrease in infectivity is due to impaired invasion as we cannot rule out other contributions such as impaired schizogony due to growth retardation or clearance from the circulation. However the latter explanation is unlikely given that it would have to be young ring-stage parasites that were being cleared. Therefore it remains open if a deficiency of ankyrin-1 in RBCs perturbs invasion of malaria parasites.

However we noticed that intraerythrocytic parasites appeared morphological abnormal in *Ank-1^MRI23420/+^* RBCs. The parasites growing in mutant cells were noticeably condensed and smaller 6–8 hours after invasion. The same phenomenon was observed at trophozoite stage (18–20 hours post-invasion). Given that this may represent parasite death, we investigated the health of *P. chabaudi* parasites *in vivo* using the TUNEL stain. The proportion of TUNEL stained dead parasites was significantly greater at ring and trophozoite stage in *Ank-1^MRI23420/+^* RBCs compared to wt. From those results we conclude that deficiency in ankyrin-1 results in increased death of the intraerythrocytic parasites which inversely affects the course of parasitemia resulting in resistance to malarial infection.

A previous study on the *P. falciparum* and human RBCs suggested that ankyrin-1 plays a role at the late stages of parasite development (Hanspal, 2002). In this process, falcipain-2 of the cysteine protease family cathepsin [49] was shown to cleave ankyrin-1 and protein 4.1 in RBCs [41]. In support another study found that several proteins associated with the RBC cytoskeleton including ankyrin-1 are proteolysed during merozoite release [50]. To demonstrate a functional role of ankyrin-1 in merozoite release, a synthetic peptide spanning the identified cleavage site of the cytoskeletal protein (amino acid 1210), prevented falcipain-2 activity and subsequent development of *P. falciparum* trophozoites into segmented schizonts and subsequent rings. Our results demonstrating a growth arrest of parasites at trophozoite stage provides *in vivo* support to their hypothesis that ankyrin-1 is important for trophozoite development.

In addition we noticed that survival of intraerythrocytic parasites was reduced at ring stage. The observation from both the TUNEL experiments and microscopic, morphological changes that parasites begin dying as early as 6 hours after invasion indicates that survival of parasites in mutant RBCs is affected before trophozoite development. This contrasts to the study from Dhawan and colleagues [40] showing that ring stage parasites incubated with the anti-ANK-1 peptide, matured to trophozoites comparably to their control group. Our observation would suggest that decreased ankyrin-1 levels affect the parasitès survival in RBCs via mechanisms additional to falcipain-2 cleavage.

Several studies have suggested that abnormal cytoskeletal proteins of RBCs may disrupt survival of intraerythrocytic parasites [15]–[17]. Pantaleo and colleagues [51] have reported that phosphorylation of several RBC membrane proteins, including band 3 and ankyrin-1, is influenced by the parasite during maturation. They speculated that the observed phosphorylative changes of cytoskeletal proteins including ankyrin-1 may be a response to perturbation of RBC homeostasis during parasite growth. However, these did not address any functional consequences of these changes. To our knowledge this is therefore the first evidence that increased resistance of *Ank-1* impaired mice is most closely related to intraerythrocytic parasite growth rates during the initial expansion in circulating parasite mass and this is consistent with a red cell autonomous effect.

In summary we generated a novel ENU-mutation in *Ank-1* resulting in an HS phenotype in mice. We demonstrated that a decrease in ankyrin-1 protein levels reduced susceptibility to malaria infections. Furthermore we revealed that *P. chabaudi* parasites display reduced growth and probably invasion and/or egress capacity in RBCs exhibiting ankyrin-1 abnormalities. The importance of ankyrin-1 during the parasites maturation stage gives us further insights into the understanding of the molecular interactions between the parasite and its host cell. This study provided also the first direct evidence that a cytoskeletal host protein plays a crucial role in parasite maturation. Further it highlights the importance of host mediated genetic resistance from cytoskeletal protein abnormalities towards malaria resistance.

## Materials and Methods

### Mice and Ethics Statement

All mice in this study were housed under controlled temperature (21±1°C) with a 12∶12 h light-dark cycle. All procedures were conducted in accordance with the policies of the University of Tasmania and conformed to the National Health and Medical Research Council (NHMRC) Australian code of Practice. The work was performed under the agreement Ethics No A0104070 approved and obtained from the Animal Ethics Committee at University of Tasmania.

### ENU Mutagenesis and Dominant Phenotype Screening

SJL/J male mice received two intraperitoneal injections of 150 mg/kg ENU (Sigma-Aldrich, Oakville, ON, Canada) at one week interval. Mutagenized SJL/J males (G0) were crossed to SJL/J females and the first generation progeny (G1) were screened for a peripheral blood (PB) phenotype from seven weeks old on an automated blood analyser (Siemens Advia 120 Automated Hematology Analyser). Mice with a “mean corpuscular volume” (MCV) parameter deviating from littermate over 3 standard deviations below the average of the G1 mice were selected and tested for their heritability through SJL/J progeny testing (G2). A student’s t-test was performed to assess statistical differences for all parameters. For genetic mapping G2 animals were backcrossed to C57BL/6 mice. F1 were screened for their microcytic MCV phenotype and affected F1 were mated with C57BL/6J strain to produce N2 generation mice that were also screened for their MCV.

### Gene Mapping

Genomic DNA was purified from the tail using the phenol chloroform extraction procedure described by Kochl *et al.* [52]. A genomewide SNP analysis on a MASSarray platform using the IPlex GLOD technology (Sequenom Inc, San Diego, CA) was conducted on 20 affected N2 mice using a mouse linkage marker set of over 600 SNPs markers including 300 polymorphic ones between C57BL/6 and SJL/J evenly distributed throughout the genome. The lod score was determined by using a chisquare test between observed values (number of heterozygous genotype mice per SNP) and theoretical values. Fine mapping of the critical interval was conducted with microsatellites genotyping of recombinant mice for chromosome 8 between 22 and 44 Mb (D8Mit170a, D8Mit95, D8Mit190, D8Mit65). Sequencing of the *Ank-1* gene was done by amplification of all exon and intron/exon boundaries using the polymerase chain reaction (PCR). For a single PCR 12.5 µL of GoTaq Green Mastermix containing Taq polymerase, magnesium chloride, DNTPs, and the loading buffer (Promega, Madison WI), 5.5 µL of distilled H_2_0, 1 µL of each specific primer (concentration 10 µmol/L), and 5 µL of the corresponding DNA sample (concentration of 20 ng/µL) were used. PCR products were purified using a DNA clean up kit (Promega Corporation, Madison WI) before sent for Sanger sequencing at the Australian Genome Research Facility (AGRF).

### SDS Page and Western Blot Analysis

For Western blotting whole blood lysate samples were separated by non-denaturing sodium dodecyl sulfate–polyacrylamide gel electrophoresis (SDS-PAGE) [53] using 8% gradient gels. For immunoblotting, samples were transferred to nitrocellulose membranes. The membrane was then incubated with either the mouse monoclonal GAPDH (Milliporo) or the N-terminal *Ank-1* antibody “p89” (kindly provided by Connie Birkenmeier, Jackson Laboratory, USA) and then washed extensively prior to incubation with peroxidase-conjugated secondary antibodies. After further washes, the blots were visualized using enhanced chemiluminescence (ECL) reagents (Amersham Biosciences, Piscataway, NJ).

Densitometry analysis was done in Photoshop CS4 (Adobe). Each band was masked containing equal amount of pixel and the mean from the histogram recorded. Values were subtracted from background and divided by the signal from corresponding GAPDH bands.

### Quantitative PCR

RNA was isolated from kidneys, spleens, brain and livers of uninfected SJL and *Ank-1^MRI23420^* heterozygous mice using the Tri-reagent (Invitrogen, Carlsbad, CA) according to the manufacturer’s recommendations. RNA was cleaned-up using the Qiagen midi kit (Qiagen, Valencia CA) and reverse transcribed with the cDNA synthesis kit (Roche-Applied Science, Basel, Switzerland) with Oligo-p(dt)_15_ primers. Quantitative PCR of cDNA was conducted using the SYBR green fluorescent dye (Roche, Basel, Switzerland) at a serial concentration from 1∶10 to 1: 160. The gene expression study was conducted on a Light Cycler 480 (Roche, Basel, Switzerland). The expression of the gene *Ank-1* was normalised to ß-Actin using the 2^−ΔΔCt^ formula and expressed as a fold change of the wild-type mice. Primers from *Ank-1* gene were spanning throughout the gene on the exons 6 and 17. The primers sequences are: *Ank-1*_1, 5′-CTACAGCAGGGTCACGAGAA-3′; *Ank-1*_1, 5′-GTCCGTGTGTCATCGTTGC-3′; *Ank-1*_2, 5′-TGCCAAGCAGAACCAGATAG-3′; *Ank-1*_2, 5′-AGTGGGGTCACGCCTTGTA-3′; ß-Actin, 5′- TTCTTTGCAGCTCCTTCGTTGCCG-3′; ß-Actin, 5′- TGGATGCGTACGTACATGGCTGGG-3′.

### Histology

Spleens and livers from uninfected SJL and *Ank-1^MRI23420/+^* mice were collected and fixed overnight in 10% formalin for 24 hours, dehydrated on the Leica ASP200 S Tissue Processor (Leica Microsystems) and embedded in paraffin wax. Sections were cut on a microtome at 5 µM and fixed to glass slides and then stained with either hematoxylin-eosin or Perl’s Prussian Blue staining.

### Total Non-heme Assay in Mouse Tissue

Liver and spleen tissue were dissected from uninfected animals. Between 50–100 mg of each tissue was dried at 45°C for 48 h hours and placed in 10% Hydrochloric acid/10% Trichloroacetic acid solution to digest for 48 hours at 65°C. Samples were then centrifuged at 13̀000 rpm for 5 minutes. 200 µl of supernatant was then added to 1 ml of 1,10-Phenanthroline monohydrate solution (Sigma-Aldrich) and incubated for 15 minutes at room temperature. After incubation 300 µl of sample was transferred to a flat bottom plate and absorbance measured at 508 nm. All samples were duplicated and for all statistical analyses a student̀s t-test was performed.

### Scanning Electron Microscopy

One to two drops of tail venous blood were sampled from uninfected and infected mice at low parasitemia (3–8%) displaying synchronised ring stage. The blood was collected in 1 ml of 1x MT-PBS (pH 7.2) and centrifuged at 500 rpm for 5 minutes at room temperature. The pellet was then fixed in 1 ml of 2.5% glutaraldehyde for 1 h under constant agitation before stored at 4°C for up to 3 days until further processing. Cells were placed on THERMANOX coverslips (ProSciTech - Queensland) and postfixed with 2.5% osmium tetroxide (OsO_4_) for 30 minutes at room temperature. Samples were rinsed 2–3 times in 10% EtOH before dehydrated through a number of incubations with increasing concentrations of ethanol (10 minute changes in each of 30, 50, 70, 80, 90, 95, 100, 100% EtOH, 100% dry EtOH, and 100% dry acetone). Thereafter samples were critical point dried with a 30 minutes purge time (tousinis autosanwdri-815) and mounted on metal stups with a carbon disk underneath. Edges were sealed with carbon paint for better conductivity and samples were sputtercoated with a 6 mm platinum layer (Cressington sputter coater 208HR with Cressington thickness controller mtm20). Imaging was carried out using a JEOL JSM-6701F scanning electron microscope.

**Figure 9:**
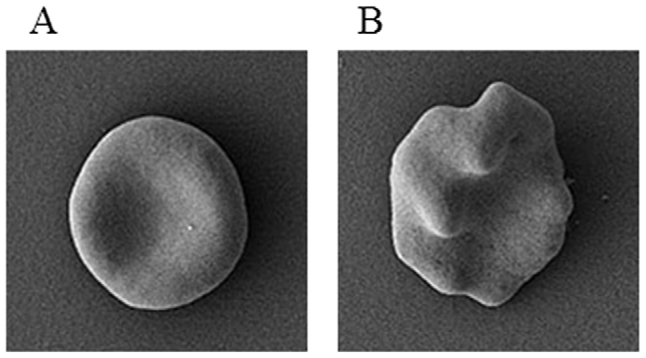
Examples of the defined morphology of RBCs in the quantification of mis-shaped RBC with SEM. (A) normal RBC and (B) abnormal RBC.

Cells were quantified by counting morphologically similar cells. Normal RBCs (Fig. 9A) were defined as displaying a classic round donut shape with an obvious centre depression and no blebs visible on the surface. Abnormal RBCs (Fig. 9B) were specified if they possessed distinct blebbing of the surface responsible for a complete loss of a round cell structure and the centre depression.

### Osmotic Fragility

Fresh blood was collected from 7 week old heterozygous and wt mice and incubated in different NaCl concentrations (from 0 to 160 mmol) for 30 min at 37°C. After gentle centrifugation, supernatant was taken and the absorbance was measured at 540 nm. The absorbance of each sample in water was recorded as 100% lysis.

### Erythropoietin Immunoassay

Blood was collected from adult uninfected wt and *Ank-1^MRI23420/+^* mice and left to clot at room temperature for 2 hours. Samples were centrifuged at 2000 g for 20 min and serum removed. A Quantikine Mouse/Rat Immunoassay (R &D Systems, Minneapolis) was used to measure the concentration of erythopoiesin (EPO) according to manufacturer instructions.

### Malaria Infection with *P. chabaudi*

The rodent malarial parasite species *P.chabaudi adami DS* was used for all experimental infections. 250 µL of thawed parasite stocks were injected into the intraperitoneal cavity of C57BL/6 donor mice. When donors reached 5–15% parasitemia, blood was collected by mandibular bleeding. An appropriate amount of blood was then diluted into 25 ml of HEM (HEPES-buffered minimum Essential Media) which consisted in Mini Essential Media (MEM) +2% of 1M HEPES and 1% Penicillin from total volume and pre-heated to 37°C to give a final parasite concentration of 1×10^4^ parasitised RBCs. Mice were monitored daily by Giesma stained blood smears from tails. Thin smears were fixed in methanol for one minute, following with ten minutes in 10% Giemsa. Parasitised RBCs were then examined under a light microscope at 100x magnification and the percentage of infected to uninfected RBCs calculated. Survival challenges were split by gender as males are known for their increased susceptibility to *P. chabaudi* infections [54], [55]. Statistical analysis for malarial survival was determined by a Mantel-Cox log rank test on a Kaplan-Meier survival curve using Prism 5.04 (Graphpad). A student̀s t-test was performed for all parasitemia screens.

For the erythrocytic parasite stage assay one drop of tail blood was sampled at 8 am and 8 pm. The assay was conducted at low parasitemia (starting at 2–4%) displaying the more likely synchronised parasites. Around 500–600 infected RBC displaying ring or trophozoite stage were counted per slide under a light microscope (magnification ×100). A separate count as described above was done for evaluating total parasitemia as described above.

### Invasion/growth in-vivo Assay

Fresh blood was collected from at least 7 weeks old uninfected wt and *Ank-1^MRI^* ^23420/+^ mice. RBCs were concentrated by centrifugation. Wt and *Ank-1^MRI^* ^23420/+^ RBCs were stained with the fluorescent dyes, ATTO 633 and 495 (Sigma Aldrich, St Louis, Missouri) in all possible combinations (wt-ATTO 633, wt-ATTO 495, *Ank1^MR23420/+^* ^-^ATTO 633 and *Ank1^MRI23420/+-^*ATTO 495). The blood was stained with the dyes according to manufacturer instructions. Stained cells were mixed into the following combinations: wt-ATTO 633 with ^Ank1MRI23420/+-^ATTO 495 and wt-ATTO 495 with *Ank1^MRI23420/+-^*ATTO 633 at a ratio of 1∶1 and diluted with 1x MT-PBS to a cell concentration of 1×10^9^ cells/mL. 0.1 mL from each blood combination was injected intraperitoneal into separated groups of infected wt and *Ank-1^MRI^* ^23420/+^ mice in the evening. The day of cell labelling and injection was chosen when parasitemia of infected mice was still similar between both cohorts at day 6 or 7 post inoculation (2–4%) as observed on giemsa stained blood smears. The next day, early morning, one drop of blood from mouse tail was collected into FACS buffer (1% BSA, 0.1% Sodium Azide in 1x MT-PBS) and stained with TER119-PE (BD Biosciences PharMingen) and Hoechst 23580. Samples were analysed using flow cytometry (FACS Canto II, BD Biosciences PharMingen). This involved gating all events for TER119+ve. From this gate the rate of double positive (ATTO 633+ Hoescht23580) out of the total ATTO-633 and the rate of the double positive (ATTO 495+ Hoescht23580) to the total number of total ATTO-495 for each recipient strain (SJL and *Ank-1* ^23420/+^) was calculated. In wt mice the combined means of wt (double positive for either TER119+ ATTO 633+ Hoescht23580 or TER119+ ATTO 495+ Hoescht23580) and *Ank-1^MRI23420/+^* (double positive for either TER119+ ATTO 633+ Hoescht23580 or TER119+ ATTO 495+ Hoescht23580) infected RBCs were expressed as 100% and the proportion of infected RBCs for each cohort were calculated. The same comparison was repeated separately in infected *Ank-1^MRI23420/+^* mice.

### Terminal Deoxynucleotidyl Transferase dUTP Nick End Labeling (TUNEL)

Blood smears from the mouse tail were fixed in 100% MeOH. For all TUNEL staining an APO-BrdU TUNEL assay kit was used (Invitrogen). Slides were initially washed 3 times with 1 ml of wash buffer solution before incubated with 50 µl of DNA labelling solution mix (according to manufacturer instructions) overnight at room temperature. The next day slides were rinsed 3 times with 1 ml rinse solution with 2 minutes incubation time each. Stain sections were then incubated with 100 µl of BrdU-antibody for 1 hour in the dark at room temperature with subsequent washes in 1%BSA/1x MT-PBS (3 times with 2 minutes soaks each). Slides were then further labelled with 100 µl of nuclear yellow (Invitrogen) (1∶5̀000 in 1% BSA/1 × MT-PBS) for 1 minute in the dark. After further washes with 1 ml MT-PBS (3 times with 2 minutes soaks each) sections were affixed with Fluorescent Mounting Medium (DakoCytomation). Once the medium dried, slides were examined on an upright epifluorescence microscope (Olympus BX50) between 600x and 1000x magnification. At least 100 nuclear yellow positive parasites present inside red blood cells were counted per slide. They were identified as having DNA fragmentation if they also stained positive for TUNEL.

### Quantification of Pyknotic Intracellular Parasites

The percentage of pyknotic vs. healthy looking intracellular parasites were calculated under a light microscope at 100x magnification. In contrast to healthy ring parasites (6–8 hour post-invasion) displaying a big (1/4–1/5 of RBC size) ring body form (Fig. 7A), ring stage parasites expressing a pyknotic phenotype (Fig. 7B) were defined if their body size is either below ≤1/4 the size of a RBC or lack a classic ring form. Pyknotic parasites 18–20 hour post-invasion (Fig. 7D) were identified if they exhibited a similar small (≤1/4 the size of a RBCs), dense, and irregular body shape lacking a clear cross-hatch or similar line pattern as opposed to healthy trophozoites (Fig. 7C).

### Statistical Analysis

If not otherwise mentioned all statistical analyses were evaluated with a Mann-Whitney-Wilcoxon test under R.2.15.0 (http://cran.r-project.org/).

## Supporting Information

Figure S1 **Gene expression level of** ***Ank-1^MRI23420/+^*** **and wild type mice in the spleen, liver, kidney and brain**. (A) *Ank-1* primers spanning the exons 6 and 7 upstream and (B) downstream (exons 17 and 18) of mutation. Error bars indicate SEM and (*) statistical differences were indicated by * with a p-value <0.05.
(TIF)

Figure S2 **H & E stained spleen tissue from uninfected wt and** ***Ank-1^MRI23420/+^*** **mice.** Spleen section from uninfected (A) wt and (B) heterozygous mice with a reduced medulla and a proliferation of the extramedullar compartment.
(TIF)

Figure S3 **Uninfected** ***Ank-1^MRI23420/+^*** **mice exhibit an iron overload phenotype in the spleen.** Histology and Perl `s blue Prussian staining for iron in (A) wt and (B) *Ank-1^MRI23420/+^* spleens. (C) Colourimetric analysis of non-heme iron in spleen and liver from uninfected *Ank-1^MRI23420/+^* and wt mice. Error bars are presented as SEM and (*) indicate statistical differences with a p-value <0.05.
(TIF)

Figure S4 **Example of flow cytometry plots corresponding to invasion/growth in-vivo assay.** (A) Quantification of infected *Ank-1^MRI23420/+^* (ATTO 633 & Hoechst 34580+ve) and wt (ATTO 495 & Hoechst 34580+ve) RBC `s in (A) *Ank-1^MRI23420/+^* and (B) wt host mice.
(TIF)

Figure S5 **Parasitemia curve between** ***Ank-1^MRI23420/+^*** **and wt mice corresponding to the TUNEL experiment.** Blood was collected from infected mice (dose 4×10^4^ iRBC) at the same time as for the time points used in the TUNEL experiment including a day after. Error bars are presented as SEM and (*) indicate statistical differences with a p-value <0.05.
(TIF)

## References

[1] Verra F, Mangano VD, Modiano D (2009). Genetics of susceptibility to Plasmodium falciparum: from classical malaria resistance genes towards genome-wide association studies.. Parasite Immunol.

[2] Sabeti PC, Schaffner SF, Fry B, Lohmueller J, Varilly P (2006). Positive natural selection in the human lineage.. Science.

[3] Haldane JB (1949). The association of characters as a result of inbreeding and linkage.. Annals of eugenics.

[4] Modiano D, Luoni G, Sirima BS, Lanfrancotti A, Petrarca V (2001). The lower susceptibility to Plasmodium falciparum malaria of Fulani of Burkina Faso (west Africa) is associated with low frequencies of classic malaria-resistance genes.. Trans R Soc Trop Med Hyg.

[5] Louicharoen C, Patin E, Paul R, Nuchprayoon I, Witoonpanich B (2009). Positively selected G6PD-Mahidol mutation reduces Plasmodium vivax density in Southeast Asians.. Science.

[6] Ko WY, Kaercher KA, Giombini E, Marcatili P, Froment A (2011). Effects of natural selection and gene conversion on the evolution of human glycophorins coding for MNS blood polymorphisms in malaria-endemic African populations.. Am J Hum Genet.

[7] Jallow M, Teo YY, Small KS, Rockett KA, Deloukas P (2009). Genome-wide and fine-resolution association analysis of malaria in West Africa.. Nature genetics.

[8] Garcia A, Cot M, Chippaux JP, Ranque S, Feingold J (1998). Genetic control of blood infection levels in human malaria: evidence for a complex genetic model.. The American journal of tropical medicine and hygiene.

[9] Wertheimer SP, Barnwell JW (1989). Plasmodium vivax interaction with the human Duffy blood group glycoprotein: identification of a parasite receptor-like protein.. Exp Parasitol.

[10] Miller LH, Mason SJ, Clyde DF, McGinniss MH (1976). The resistance factor to Plasmodium vivax in blacks. The Duffy-blood-group genotype, FyFy.. N Engl J Med.

[11] Horuk R, Chitnis CE, Darbonne WC, Colby TJ, Rybicki A (1993). A receptor for the malarial parasite Plasmodium vivax: the erythrocyte chemokine receptor.. Science.

[12] Tournamille C, Colin Y, Cartron JP, Le Van Kim C (1995). Disruption of a GATA motif in the Duffy gene promoter abolishes erythroid gene expression in Duffy-negative individuals.. Nature genetics.

[13] Mohandas N, Gallagher PG (2008). Red cell membrane: past, present, and future.. Blood.

[14] Rank G, Sutton R, Marshall V, Lundie RJ, Caddy J (2009). Novel roles for erythroid Ankyrin-1 revealed through an ENU-induced null mouse mutant.. Blood.

[15] Schulman S, Roth EF, Cheng B, Rybicki AC, Sussman, II, et al (1990). Growth of Plasmodium falciparum in human erythrocytes containing abnormal membrane proteins.. Proc Natl Acad Sci U S A.

[16] Shear HL (1993). Transgenic and mutant animal models to study mechanisms of protection of red cell genetic defects against malaria.. Experientia.

[17] Shear HL, Roth EF, Ng C, Nagel RL (1991). Resistance to malaria in ankyrin and spectrin deficient mice.. Br J Haematol.

[18] Zimmerman PA, Patel SS, Maier AG, Bockarie MJ, Kazura JW (2003). Erythrocyte polymorphisms and malaria parasite invasion in Papua New Guinea.. Trends Parasitol.

[19] Chishti AH, Palek J, Fisher D, Maalouf GJ, Liu SC (1996). Reduced invasion and growth of Plasmodium falciparum into elliptocytic red blood cells with a combined deficiency of protein 4.1, glycophorin C, and p55.. Blood.

[20] Gallagher PG (2005). Hematologically important mutations: ankyrin variants in hereditary spherocytosis.. Blood Cells Mol Dis.

[21] Cooke BM, Mohandas N, Coppel RL (2004). Malaria and the red blood cell membrane.. Semin Hematol.

[22] Maier AG, Cooke BM, Cowman AF, Tilley L (2009). Malaria parasite proteins that remodel the host erythrocyte.. Nat Rev Microbiol.

[23] Berghout J, Min-Oo G, Tam M, Gauthier S, Stevenson MM (2010). Identification of a novel cerebral malaria susceptibility locus (Berr5) on mouse chromosome 19.. Genes and immunity.

[24] Hernandez-Valladares M, Naessens J, Iraqi FA (2005). Genetic resistance to malaria in mouse models.. Trends in parasitology.

[25] Longley R, Smith C, Fortin A, Berghout J, McMorran B (2011). Host resistance to malaria: using mouse models to explore the host response.. Mamm Genome.

[26] Ohno T, Ishih A, Kohara Y, Yonekawa H, Terada M (2001). Chromosomal mapping of the host resistance locus to rodent malaria (Plasmodium yoelii) infection in mice.. Immunogenetics.

[27] Carvalho LJ (2010). Murine cerebral malaria: how far from human cerebral malaria?. Trends in parasitology.

[28] Hunt NH, Grau GE, Engwerda C, Barnum SR, van der Heyde H (2010). Murine cerebral malaria: the whole story.. Trends in parasitology.

[29] Yap GS, Stevenson MM (1992). Plasmodium chabaudi AS: erythropoietic responses during infection in resistant and susceptible mice.. Exp Parasitol.

[30] Fortin A, Belouchi A, Tam MF, Cardon L, Skamene E (1997). Genetic control of blood parasitaemia in mouse malaria maps to chromosome 8.. Nature genetics.

[31] Foote SJ, Burt RA, Baldwin TM, Presente A, Roberts AW (1997). Mouse loci for malaria-induced mortality and the control of parasitaemia.. Nature genetics.

[32] Burt RA, Marshall VM, Wagglen J, Rodda FR, Senyschen D (2002). Mice that are congenic for the char2 locus are susceptible to malaria.. Infection and immunity.

[33] Lin E, Pappenfuss T, Tan RB, Senyschyn D, Bahlo M (2006). Mapping of the Plasmodium chabaudi resistance locus char2.. Infect Immun.

[34] Yates L, McMurray F, Zhang Y, Greenfield A, Moffatt M (2009). ENU mutagenesis as a tool for understanding lung development and disease.. Biochem Soc Trans.

[35] Aigner B, Rathkolb B, Herbach N, Hrabe de Angelis M, Wanke R (2008). Diabetes models by screen for hyperglycemia in phenotype-driven ENU mouse mutagenesis projects.. Am J Physiol Endocrinol Metab.

[36] Crozat K, Georgel P, Rutschmann S, Mann N, Du X (2006). Analysis of the MCMV resistome by ENU mutagenesis.. Mamm Genome.

[37] Hoebe K, Beutler B (2008). Forward genetic analysis of TLR-signaling pathways: an evaluation.. Adv Drug Deliv Rev.

[38] Richer E, Prendergast C, Zhang DE, Qureshi ST, Vidal SM (2010). N-ethyl-N-nitrosourea-induced mutation in ubiquitin-specific peptidase 18 causes hyperactivation of IFN-alphass signaling and suppresses STAT4-induced IFN-gamma production, resulting in increased susceptibility to Salmonella typhimurium.. J Immunol.

[39] Eber SW, Gonzalez JM, Lux ML, Scarpa AL, Tse WT (1996). Ankyrin-1 mutations are a major cause of dominant and recessive hereditary spherocytosis.. Nature genetics.

[40] Dhawan S, Dua M, Chishti AH, Hanspal M (2003). Ankyrin peptide blocks falcipain-2-mediated malaria parasite release from red blood cells.. J Biol Chem.

[41] Hanspal M, Dua M, Takakuwa Y, Chishti AH, Mizuno A (2002). Plasmodium falciparum cysteine protease falcipain-2 cleaves erythrocyte membrane skeletal proteins at late stages of parasite development.. Blood.

[42] Raphael P, Takakuwa Y, Manno S, Liu SC, Chishti AH (2000). A cysteine protease activity from Plasmodium falciparum cleaves human erythrocyte ankyrin.. Mol Biochem Parasitol.

[43] Hughes MR, Anderson N, Maltby S, Wong J, Berberovic Z (2011). A novel ENU-generated truncation mutation lacking the spectrin-binding and C-terminal regulatory domains of Ank1 models severe hemolytic hereditary spherocytosis.. Experimental hematology 39: 305–320, 320 e301–302.

[44] Randon J, Miraglia del Giudice E, Bozon M, Perrotta S, De Vivo M (1997). Frequent de novo mutations of the ANK1 gene mimic a recessive mode of transmission in hereditary spherocytosis: three new ANK1 variants: ankyrins Bari, Napoli II and Anzio.. British journal of haematology.

[45] Chimanuka B, Vanden Driessche T, Lisgarten JN, Plaizier-Vercammen J (1999). Influence of the Host Photoperiodicity on the Schizogonic Cycle and the Synchronism of the Rodent Malaria Plasmodium chabaudi chabaudi.. Biological Rhythm Research.

[46] McMorran BJ, Marshall VM, de Graaf C, Drysdale KE, Shabbar M (2009). Platelets kill intraerythrocytic malarial parasites and mediate survival to infection.. Science.

[47] Gallagher PG, Forget BG (1998). Hematologically important mutations: spectrin and ankyrin variants in hereditary spherocytosis.. Blood Cells Mol Dis.

[48] Birkenmeier CS, Gifford EJ, Barker JE (2003). Normoblastosis, a murine model for ankyrin-deficient hemolytic anemia, is caused by a hypomorphic mutation in the erythroid ankyrin gene Ank1.. Hematol J.

[49] Hanspal M (2000). cDNA cloning of a novel cysteine protease of Plasmodium falciparum.. Biochim Biophys Acta.

[50] Millholland MG, Chandramohanadas R, Pizarro A, Wehr A, Shi H (2011). The malaria parasite progressively dismantles the host erythrocyte cytoskeleton for efficient egress.. Mol Cell Proteomics.

[51] Pantaleo A, Ferru E, Carta F, Mannu F, Giribaldi G (2010). Analysis of changes in tyrosine and serine phosphorylation of red cell membrane proteins induced by P. falciparum growth.. Proteomics.

[52] Kochl S, Niederstatter H, Parson W (2005). DNA extraction and quantitation of forensic samples using the phenol-chloroform method and real-time PCR.. Methods Mol Biol.

[53] Laemmli U (1970). Cleavage of structural proteins during the assembly of the head of bacteriophage T4.. Nature.

[54] Stevenson MM, Lyanga JJ, Skamene E (1982). Murine malaria: genetic control of resistance to Plasmodium chabaudi.. Infect Immun.

[55] Stevenson MM, Tam MF, Rae D (1990). Dependence on cell-mediated mechanisms for the appearance of crisis forms during Plasmodium chabaudi AS infection in C57BL/6 mice.. Microb Pathog.

