# Supplementary figures and images for: A Novel ENU-Mutation in Ankyrin-1 Disrupts Malaria Parasite Maturation in Red Blood Cells of Mice
Source: PLoS One. 2012 Jun 19;7(6):e38999. doi: 10.1371/journal.pone.0038999 (PMC3378575; doi:10.1371/journal.pone.0038999)

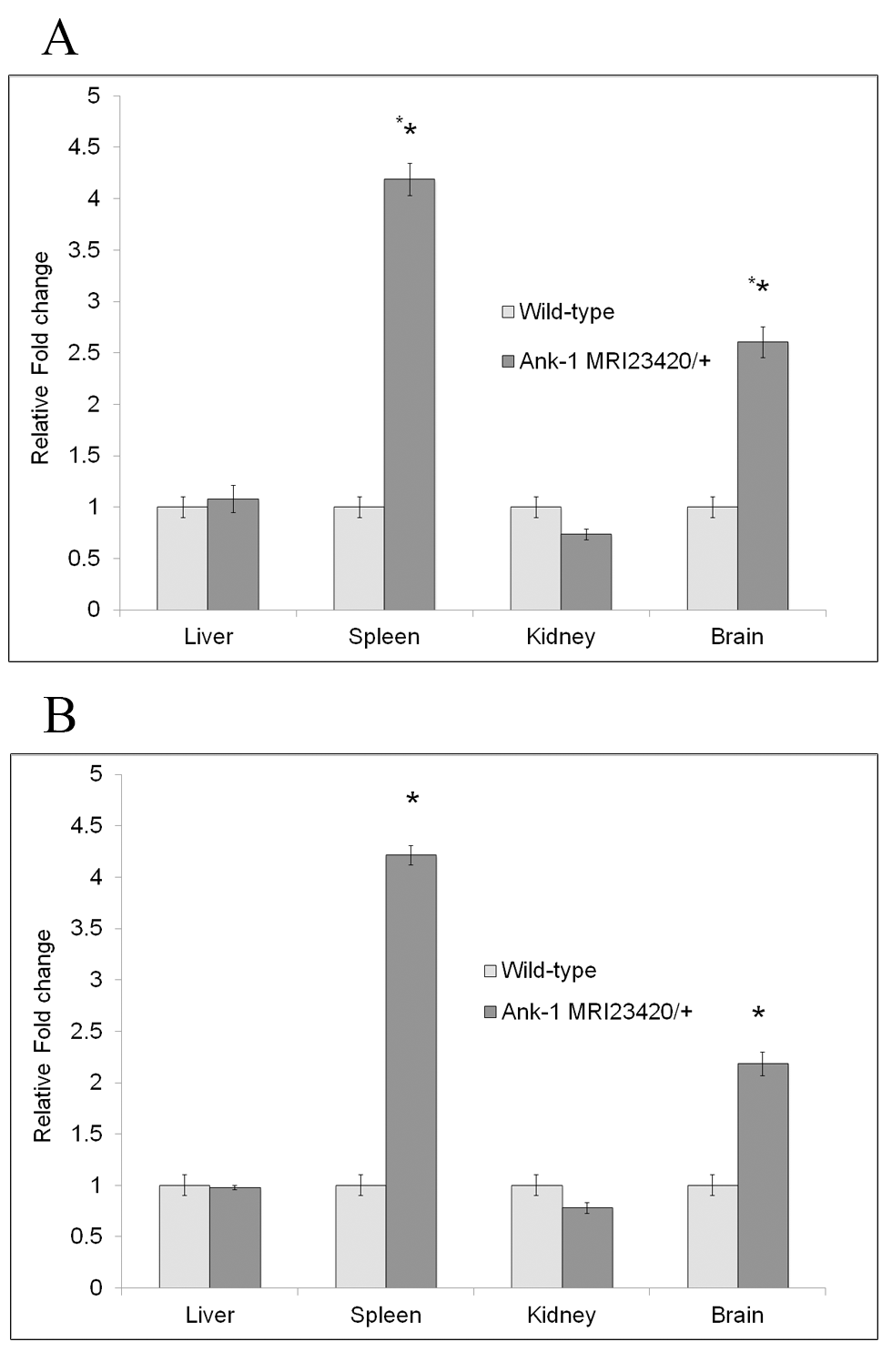

Supplement: Figure S1 — Gene expression level of Ank-1MRI23420/+ and wild type mice in the spleen, liver, kidney and brain. (A) Ank-1 primers spanning the exons 6 and 7 upstream and (B) downstream (exons 17 and 18) of mutation. Error bars indicate SEM and (*) statistical differences were indicated by * with a p-value <0.05. (TIF) [file pone.0038999.s001.tif]

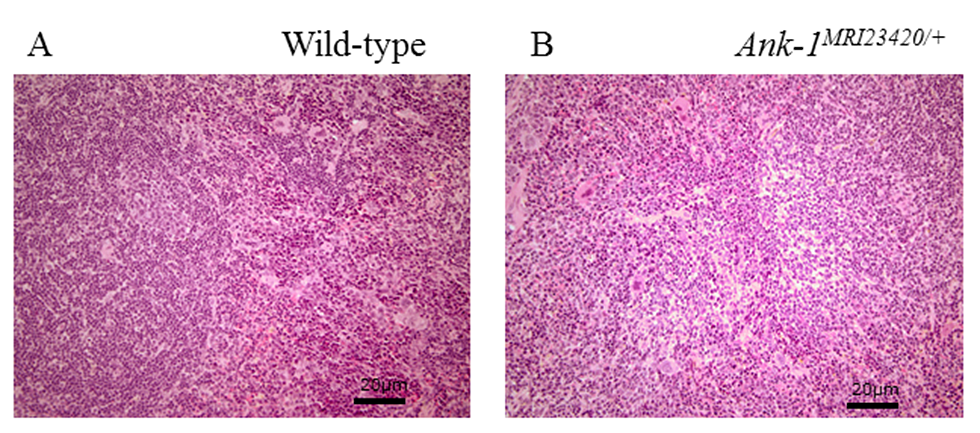

Supplement: Figure S2 — H & E stained spleen tissue from uninfected wt and Ank-1MRI23420/+ mice. Spleen section from uninfected (A) wt and (B) heterozygous mice with a reduced medulla and a proliferation of the extramedullar compartment. (TIF) [file pone.0038999.s002.tif]

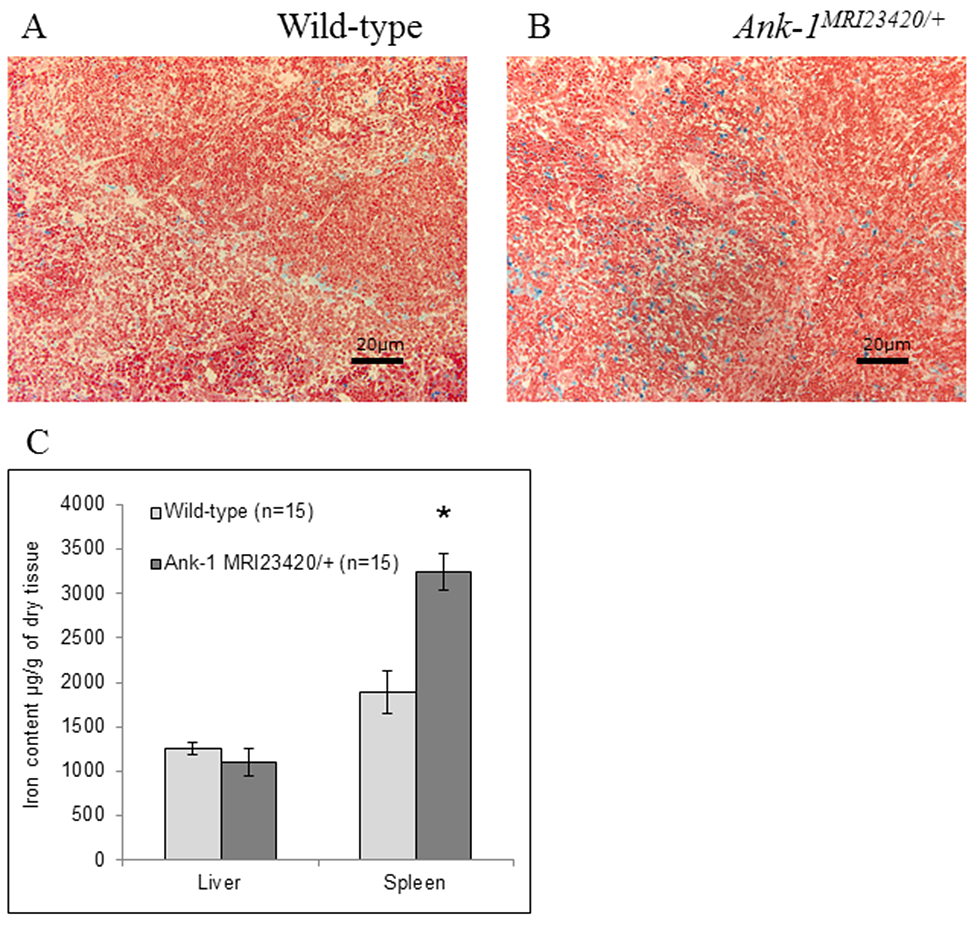

Supplement: Figure S3 — Uninfected Ank-1MRI23420/+ mice exhibit an iron overload phenotype in the spleen. Histology and Perl `s blue Prussian staining for iron in (A) wt and (B) Ank-1MRI23420/+ spleens. (C) Colourimetric analysis of non-heme iron in spleen and liver from uninfected Ank-1MRI23420/+ and wt mice. Error bars are presented as SEM and (*) indicate statistical differences with a p-value <0.05. (TIF) [file pone.0038999.s003.tif]

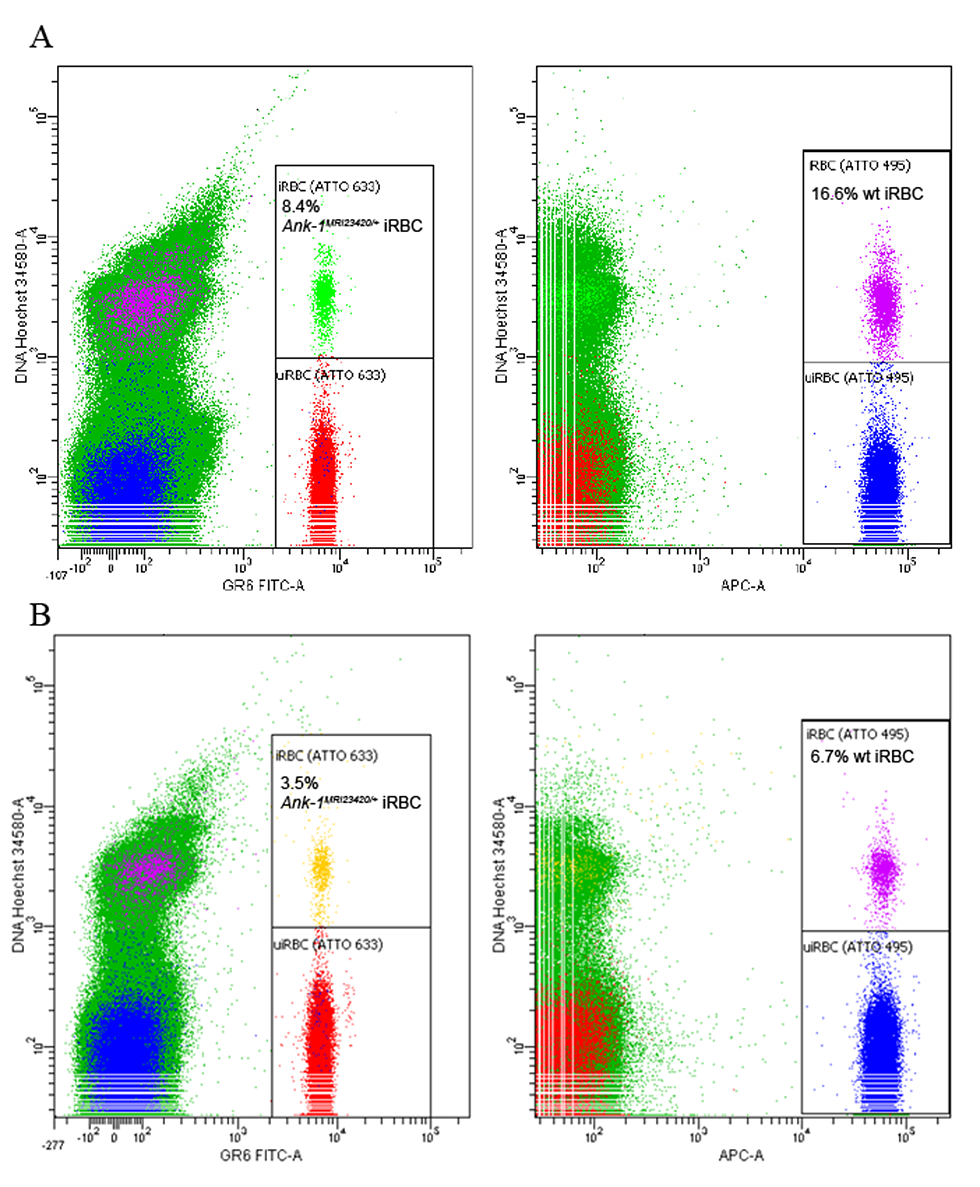

Supplement: Figure S4 — Example of flow cytometry plots corresponding to invasion/growth in-vivo assay. (A) Quantification of infected Ank-1MRI23420/+ (ATTO 633 & Hoechst 34580+ve) and wt (ATTO 495 & Hoechst 34580+ve) RBC `s in (A) Ank-1MRI23420/+ and (B) wt host mice. (TIF) [file pone.0038999.s004.tif]

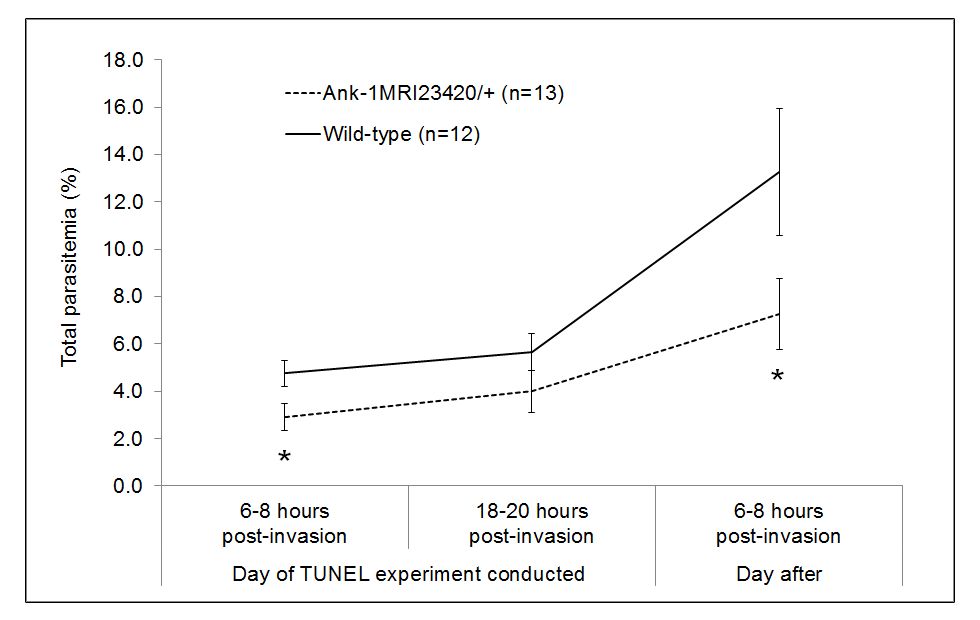

Supplement: Figure S5 — Parasitemia curve between Ank-1MRI23420/+ and wt mice corresponding to the TUNEL experiment. Blood was collected from infected mice (dose 4×10^4^ iRBC) at the same time as for the time points used in the TUNEL experiment including a day after. Error bars are presented as SEM and (*) indicate statistical differences with a p-value <0.05. (TIF) [file pone.0038999.s005.tif]
